# Brazing of Mo to Glidcop Dispersion Strengthened Copper for Accelerating Structures

**DOI:** 10.3390/ma11091658

**Published:** 2018-09-07

**Authors:** Valentina Casalegno, Sergio Perero, Monica Ferraris, Mauro Taborelli, Gonzalo Arnau Izquierdo, Stefano Sgobba, Milena Salvo

**Affiliations:** 1 Applied Science and Technology Department, Institute of Materials Physics and Engineering, Politecnico di Torino, I-10129 Torino, Italy; sergio.perero@polito.it (S.P.); monica.ferraris@polito.it (M.F.); milena.salvo@polito.it (M.S.); 2 CERN European Organization of Nuclear Research, CH-1211 Geneva, Switzerland; Mauro.Taborelli@cern.ch (M.T.); Gonzalo.Arnauizquierdo@cern.ch (G.A.I.); Stefano.Sgobba@cern.ch (S.S.)

**Keywords:** brazing, alumina dispersion-strengthened copper, mechanical testing

## Abstract

Alumina dispersion-strengthened copper, Glidcop, is used widely in high-heat-load ultra-high-vacuum components for synchrotron light sources (absorbers), accelerator components (beam intercepting devices), and in nuclear power plants. Glidcop has similar thermal and electrical properties to oxygen free electrical (OFE) copper, but has superior mechanical properties, thus making it a feasible structural material; its yield and ultimate tensile strength are equivalent to those of mild-carbon steel. The purpose of this work has been to develop a brazing technique to join Glidcop to Mo, using a commercial Cu-based alloy. The effects of the excessive diffusion of the braze along the grain boundaries on the interfacial chemistry and joint microstructure, as well as on the mechanical performance of the brazed joints, has been investigated. In order to prevent the diffusion of the braze into the Glidcop alloy, a copper barrier layer has been deposited on Glidcop by means of RF-sputtering.

## 1. Introduction

Glidcop is a family of dispersion strengthened copper alloys, made of a pure copper matrix strengthened by a uniform dispersion of alumina particles. These particles, which are stable at high temperatures and above the melting point of the matrix, have the aim of preventing the softening and recrystallization of the copper when treated at high temperatures [1]. Glidcop exhibits an improved mechanical resistance, especially at high temperatures, with respect to copper, and at the same time it maintains an excellent thermal conductivity. For these reasons, it has been considered, from the very beginning, as a suitable support material for the collimator jaws of modern particle accelerators [2], with the idea that the Glidcop block could be efficiently cooled and would work as a heat sink for the jaw itself. In this sense, it is still the material of choice for the next generation supports of the collimators [3] that have to be installed on the Large Hadron Collider (LHC) during the long shutdown 2. The jaws close to the Glidcop holder are made of a sequence of materials with an increasing atomic number, so that the extremely high energetic proton beam halo is gradually dispersed and finally absorbed. As a consequence, refractory materials, such as tungsten, molybdenum, inermet or similar, are used. In this sense, an effective joint between a highly conducting material and a high Z material could be of interest. The present work focuses on the development of a brazing process for pure Mo and a Glidcop alloy. The authors have already investigated the joining of a CuZr alloy (UNS C15000, 80% cold worked) and Mo in the framework of the Compact Linear Collider (CLIC) project [4]; the accelerating structure is traditionally made of brazed oxygen free electrical (OFE) copper parts, but CuZr and Glidcop could represent improved alternatives for the conducting regions that suffer from to mechanical fatigue. Moreover, the study of the joining of Glidcop can be considered relevant, not only for the particle accelerator components, but also for other application fields, such as resistance welding electrodes, incandescent light bulb leads, hybrid circuit packages and other high temperature applications, such as X-ray tube components or heat exchanger components [5,6]. 

Several methods have been proposed to join Glidcop to such metals as Cu or stainless steel [7,8,9,10]. An assembly of Glidcop cooling pipes, coated with a sacrificial armor in pure W or W alloys has been obtained for the diverter component using TiCuAg alloy as the joining material [6], but to the best of the authors’ knowledge, no joint between Glidcop and Mo has yet been manufactured or characterized. Of all the different joining technologies, the brazing process is known to play the most important role in the joining of Glidcop. Moreover, fusion welding, including electron beam welding, is not suitable for the joining process because the remelting of the copper matrix leads to an agglomeration of the alumina particles and recrystallization of the matrix in the welding area, thus creating a brittle welded zone [11].

The brazing of Glidcop is usually carried out with the brazing filler metals that are commonly used to join plain copper (i.e., gold- and silver-based braze alloys). The brazing of copper generally leads to a grain coarsening, due to the grain growth that occurs during the brazing process. Glidcop does not suffer from this problem, because of its fine structure and reduced recrystallization at the brazing temperatures. 

The main problem related to the brazing of Glidcop is the excessive diffusion of the silver-based filler along the grain boundaries of Glidcop. The electroplating of Glidcop with copper or nickel prior to brazing is used as a common method to prevent and solve this issue [10]. Copper plating is usually carried out in a copper cyanide solution; the cyanide-copper bath has the aim of facilitating the quality of the braze joints by preventing diffusion of the braze alloy into the Glidcop base material. On the other hand, the Cu-plating process introduces additional steps in the manufacturing process, since additional control stages are needed to ensure blister-free plating. Nickel plating is obtained through more complicated processes, which are based on a Watts bath or electroplating.

The purpose of this work has been to develop a brazing technique to join Glidcop to Mo using a commercial silver-free Cu-based alloy. In order to prevent the diffusion of the braze into the Glidcop, a copper barrier layer has been deposited on the Glidcop by means of RF-sputtering, an environmentally friendly coating technique, which does not require the use of any hazardous chemicals.

## 2. Materials and Methods

Glidcop is a metal matrix composite made up of oxygen free copper and aluminum oxide; it consists of more than 99% copper. The Glidcop Grade used in this work is Glidcop AL-25, in which the aluminum oxide content is 0.5 wt.%; the commercial name is UNS-C15725, and the Al_2_O_3_ submicroscopic dispersed particle size ranges from 3 to 12 nm; it was supplied by Höganäs AB (Höganäs, Sweden), while the Mo (99.97% purity) was supplied by Plansee (Gemeinde Reutte, Austria).

The joining process between Glidcop and Mo was performed using a silver-free commercial metal braze (Gemco), which does not contain elements such as Ti, Si, etc. that could lead to the formation of brittle intermetallics with Cu. The braze was supplied by Wesgo Metals, and it is composed of 87.75 wt.% Cu, 12 wt.% Ge and 0.25 wt.% Ni; it has a liquidus temperature at 975 °C and a solidus temperature at 880 °C. The braze foil thickness is about 60 µm.

The wettability of the brazing alloy on the Glidcop alloy was measured by means of a hot stage microscope (model AII, Leitz GmbH, Wetzlar, Germany), up to 1000 °C under flowing Ar, at a heating rate of 20 °C·min^−1^, equipped with a Leica DBP camera (Ernst Leitz GMBH, Wetzlar, Germany).

The adherends were cut using a diamond-grinding disc, and this was followed by grinding with 2500 grit SiC paper. The final polishing was performed using a 3 µm diamond paste. The samples were then cleaned in an ultrasonic bath with acetone for 5 min to remove surface impurities. A Cu coating was deposited on the Glidcop plates for a certain set of samples, by means of Radio Frequency magnetron sputtering, before the brazing process. A copper target (99.99% purity), supplied by Franco Corradi (Rho (MI), Italy), was used. 

The sputtering deposition parameters were varied in order to optimize the homogeneity of the coating. A pure Ar (99.996% purity) atmosphere was used to avoid oxidization of the sputtered layer during deposition. 

The cathode-substrate distance was maintained fixed at 14 cm. The pre-deposition pressure reached 7.0 × 10^−5^ Pa, while the Ar pressure was 55 × 10^−1^ Pa during deposition.

A power of 250 W was applied in RF (radio frequency), to a 6 inch diameter target. This value was selected, after some preliminary depositions, in order to balance the deposition rate, which needed to be as high as possible, and the thin film morphology. Low power can lead to voids and defects in the layer, while high power can create other unwanted structures, such as columns and recrystallization. The film thickness was controlled while varying the deposition time. Different time periods (from 20 min to 7 h) were considered to obtain Cu coatings of various thickness (from 1 µm to 18 µm). The thickness measurements were performed by means of a surface profiler (Tenkor P11, KLA-Tenkor, Milpitas, CA, USA). The homogeneity area of the deposition surface was around 100 mm^2^.

The produced joints were sandwich-like Mo/Gemco/Glidcop. The metallic parts that had to be joined and the brazing alloy foil were sectioned into 5 mm × 10 mm pieces, to obtain a joining surface of 50 mm^2^. One foil or three foils of the Gemco brazing alloy were used. The brazing was performed in a horizontal tube furnace (BICASA, Camera SUPERTHAL, Bernareggio (MB), Italy) under flowing Ar; the brazing temperature was chosen slightly above the filler metal liquidus temperature (980 °C), and the heating rate adopted to reach the brazing temperature was 1000 °C·h^−1^. The dwelling time was varied from 1 min to 30 min; the specimens were then allowed to cool to room temperature in the furnace (cooling rate of 5 °C min^−1^). The influence of a tungsten weight (100 g, corresponding to 0.02 MPa nominal pressure) on the top of the Mo/Glidcop sandwich structure was also investigated. This applied load is about ten times the specimens’ own mass. 

The microstructure of the joined samples was investigated by means of optical and electron microscopy (SEM, Philips 525 M, FEI_thermo Fisher Scientific, Hillsboro, OR, USA, accelerating voltage = 15–30 kV) coupled with energy dispersive spectroscopy for the compositional analysis (EDS SW9100 EDAX and Oxford Isis 300, EDAX-AMETEK Materials Analysis Division, Meerbusch, Germany).

**Figure 1:**
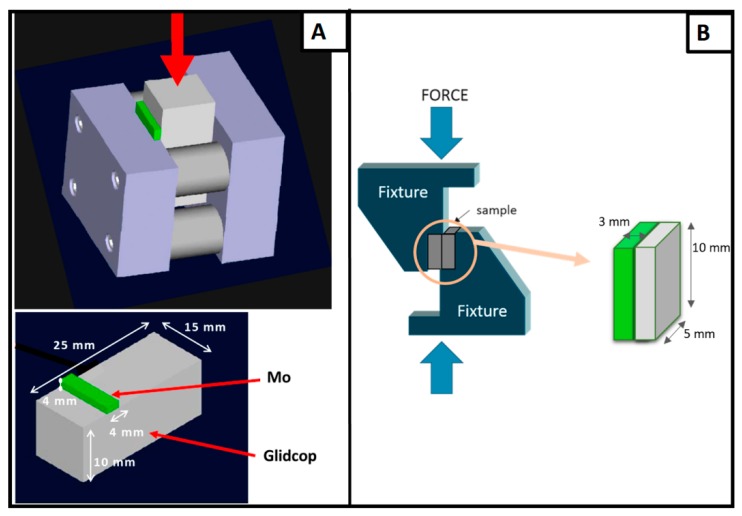
Configuration for measuring the apparent shear strength of Mo/Glidcop joined samples: (**A**) ASTM B898-11 standard (Glidcop size of 25 × 15 × 10 mm and Mo size 4 × 15 × 4 mm) and (**B**) single-lap test, adapted from ASTM D905-08 (adherends size: 5 × 10 × 3 mm).

The apparent shear strength of the joints was measured with a single-lap test under compression, adapted from ASTM D905-08 standard [12], specimen size: 5 × 10 × 3 mm, seen in Figure 1B, and according the ASTM B898-11 standard [13] (Glidcop size of 25 × 15 × 10 mm and Mo size of 4 × 15 × 4 mm), seen in Figure 1A, at room temperature (universal testing machine SINTEC D/10, MTS Systems, Eden Prairie, Minnesota, USA). The shear strength was determined as the ratio of the load measured at the fracture and the joined area. The compressive force was applied at a speed of 0.5 mm·s^−1^. The fracture surfaces were examined to determine the fracture propagation. Rockwell B hardness measurements were performed on the Glidcop surface, before and after the brazing process, using an Officine Galileo hardness tester (Durometro Galileo A200, Rockwell, Officine Galileo, Capalle (FI), Italy).

## 3. Results and Discussion

**Figure 2:**
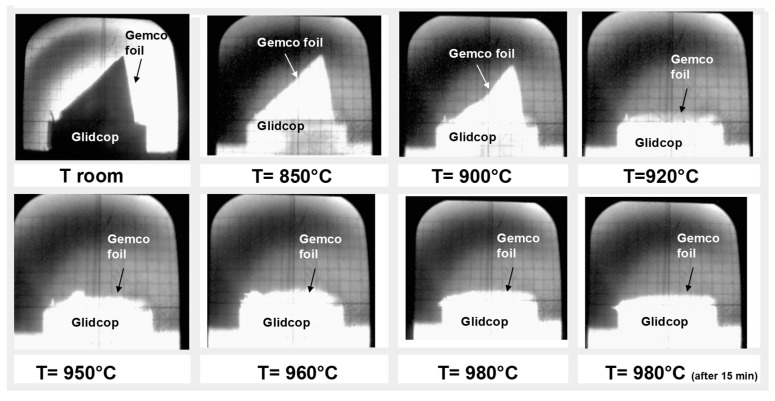
Hot stage microscope images of a Gemco braze foil on Glidcop substrate; the best spreadability was reached at 980 °C (under flowing Ar, heating rate 20 °C·min^−1^).

The wettability of the brazing alloy was only measured on the Glidcop substrate, in order to demonstrate the compatibility and spreading of the braze on Glidcop; the braze wettability on Mo had already been presented and discussed elsewhere [4]; moreover, the wetting of the Cu-Mo system is a well-known and broadly studied topic [14]. The wettability test showed that the molten alloy starts spreading at 920 °C and at 980 °C the contact angle between the molten Gemco and Glidcop reaches its lowest value, as seen in Figure 2. An equilibrium configuration is reached almost immediately, that is, after one min at 980 °C; after 15 min of contact, no variation in the spreading behavior was observed. 

As far as the brazing process is concerned, a preliminary study was carried out to optimize the thickness of the joint and its microstructure. The joint gap was controlled by the thickness of the brazing filler foil; a reduction in the joint thickness can be helpful to obtain a joint with good electrical properties [4]. The brazing treatment was performed at 980 °C, that is, at a temperature slightly above the liquidus of the filler alloy, in order to reach the best spreadability and wettability of the braze and to avoid the recovery and the softening of the copper alloy [8,9]. 

**Figure 3:**
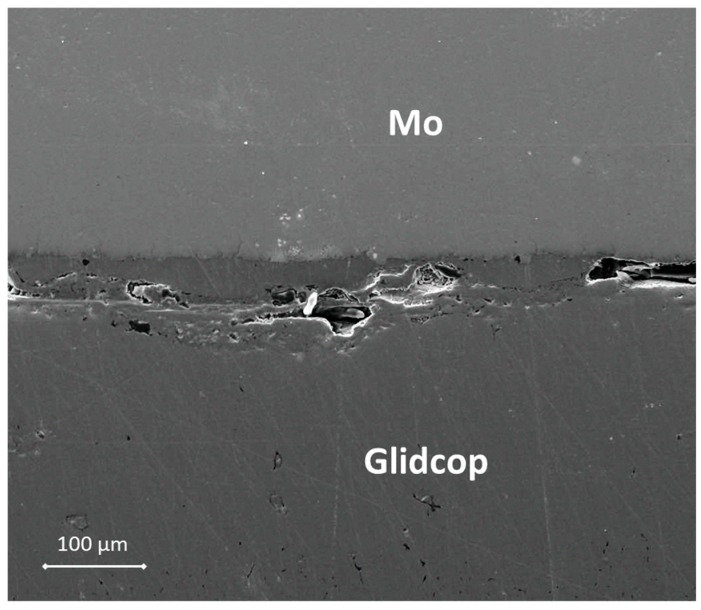
SEM micrograph of the cross-section of a Mo/Gemco/Glidcop joint manufactured by using one Gemco braze foil at 980 °C, 5 min, under flowing Ar and applying 0.02 MPa.

Figure 3 shows a SEM cross-section of an Mo/Gemco/Glidcop joint obtained using one braze foil (at 980 °C for 5 min, under flowing Ar and applying 0.02 MPa).

**Figure 4:**
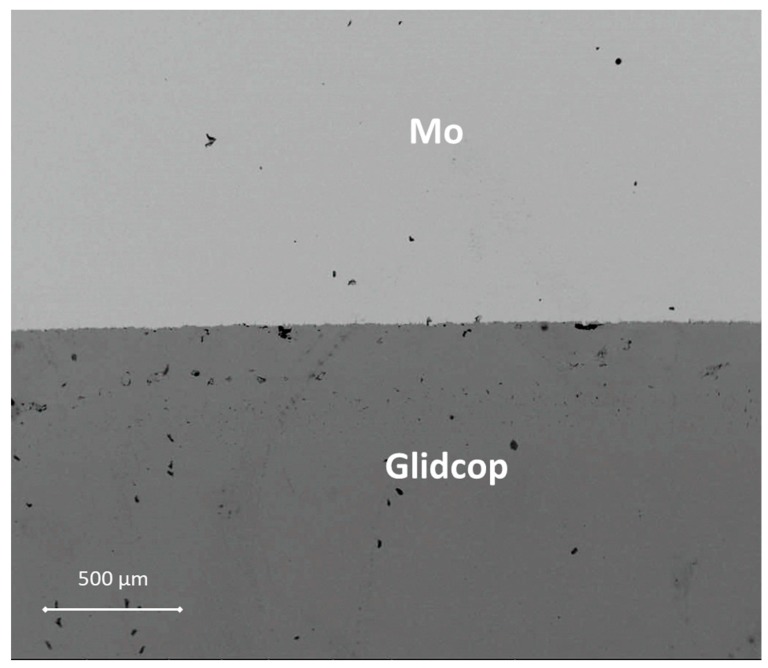
SEM micrograph of the cross-section of a Mo/Gemco/Glidcop joint manufactured by using three Gemco braze foils at 980 °C, 5 min, under flowing Ar and applying 0.02 MPa.

The thickness of the braze metal, after the thermal treatment in the samples joined with one brazing alloy foil, was about 40 μm. Because of the high wettability of the brazing alloy on the substrates, the braze can flow out of the joint gap, thus justifying the reduction in braze thickness. Several voids in the filler metal layer were visible and the interface was not continuous. On the other hand, there is little evidence of significant defects, such as micro-voids, in the joints manufactured using three Gemco braze foils, shown in Figure 4, due to braze solidification shrinkage, and both Mo and Glidcop exhibit a good metallurgical continuity in the interfacial region. Moreover, a comparison between two Mo/Glidcop joints obtained with three Gemco foils at 980 °C, 5 min, but with and without a superimposed load (100 g, corresponding to 0.02 MPa nominal pressure), was carried out. When the pressure was not applied to the assembly, a higher number of voids developed, due to the shrinkage of the liquid braze (pictures not reported here); then, the introduction of an external load during the brazing process was able to reduce these voids. From a qualitative observation of the cross-section of the samples, the surface corresponding to voids is reduced by 80%. Further investigations were addressed to optimize the dwelling time; all the different experimental parameters are summarized in Table 1. 

In order to study the distribution of the elements across the joint region, EDS analyses were performed on the Mo/Gemco/Glidcop samples brazed using three braze foils (best morphological result, according to the microstructure shown in Figure 4) at 980 °C for 1, 5, 10, 15, 30 min and applying 0.02 MPa (pictures not reported here). Germanium diffused extensively from the braze to the Glidcop substrate in all the samples. The Ni content could not be determined because its concentration was too low (0.25 wt.% of Ni in the brazing alloy). 

**Figure 5:**
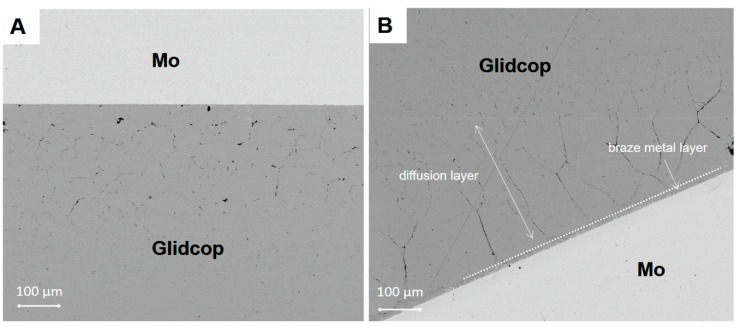
SEM micrographs of the cross-section of a Glidcop/Mo interface obtained by means of brazing (three Gemco braze foils) at 980 °C for 5 min (**A**) and for 30 min (**B**) under flowing Ar and applying 0.02 MPa.

Further investigations, by means of SEM, allowed an excessive diffusion of the braze to be detected along the grain boundaries of the Glidcop alloy, as it is possible to observe in the micrographs reported in Figure 5 for samples brazed at 980 °C for both 5 and 30 min using three Gemco alloy foils and applying 0.02 MPa. 

This result is consistent with the common diffusion theory and literature references. Several studies [15] have reported that, during the brazing of Glidcop with gold or silver alloys (mainly Au-Cu or Ag-Cu based alloys), there is a tendency of the gold or silver to diffuse into the Glidcop, depending on the duration of the brazing cycle. To the best of the authors’ knowledge, no studies on Ge and Ni diffusion in DS-copper alloys are available in literature.

The brazed area can be divided into the braze metal layer and the diffusion layer, in which the liquid metal migrates along the Glidcop grain boundaries, as indicated in Figure 5. In the BSE-SEM images shown in Figure 5, the braze was found to migrate along the Glidcop grain boundaries for several hundreds of microns, thus determining the formation of a significant porosity in the Glidcop alloy; there is evidence that the number of voids at the grain boundaries increases slightly and the voids grow in size (qualitative observation, void ratio/size not investigated) on the basis of the brazing process dwelling time (the effect of the temperature has not been studied here). This phenomenon can be explained by considering the coalescence of the pre-existing voids at the grain boundary or by the depletion of elements (i.e., Ge) from the brazing alloy that penetrates the grain boundaries. According to the Ge-Cu phase diagram [16], the Gemco alloy is at a completely liquid state at the brazing temperature, and this allows the formation of a thick diffusion layer. Only the diffusion layer can be detected from the morphological analysis, and it clearly exceeds the expected filler metal layer thickness (about 180 µm). According to Figure 5, the diffusion distance in Glidcop/Mo joints obtained using three Gemco braze foils at 980 °C for 5 min and for 30 min is about 250 µm and 350 µm, respectively. As in the case of silver-based brazes, the rate of diffusion of Gemco along the grain boundaries of Glidcop is higher than its rate of diffusion through the grains; as a consequence, there is a preferential diffusion or migration of Gemco along the grain boundaries and a successive formation of voids at the grain boundaries. Conversely, the diffusion rate of the elements contained in the brazing alloys along the grain boundaries is significant in Glidcop, and this allows a rapid migration of the elements inside the materials and a depletion of the joining area. 

It has been reported that fine grained Glidcop, which have several grain boundaries, permits a rapid diffusion of the constituents of the braze alloy, particularly Ag, along the grain boundaries [7]. Moreover, this excessive diffusion of elements along the grain boundaries of Glidcop causes the formation of small voids near the braze area. Therefore, in order to limit the diffusion phenomena of Ge and Cu, the dwelling time was reduced to 1 min; as a consequence, the joining process was carried out at 980 °C, for only 1 min, using three braze layers and applying 0.02 MPa. The morphological analysis (not reported here) of the cross section of the Mo/Glidcop joints manufactured according to the aforementioned parameters does not differ significantly from that observed for the 5 min dwelling time for the brazing process, as seen in Figure 4. Consequently, to overcome the braze diffusion along the Glidcop grain boundaries, the Glidcop surface was coated with pure Cu prior to the brazing process.

Copper and nickel coatings are well known barrier layers to the diffusion of metallic elements into Glidcop; the minimum required coating thickness for a brazing treatment is usually a function of the brazing temperature and the brazing time.

Furthermore, the adherence of the coating to the substrate plays an important role. A Watts bath or nickel sulphamate bath are generally used for the nickel plating, and the grain size and orientation of the plated layers can influence the barrier function of the plating. A cyanide bath or an acid copper sulfate bath are currently used for copper plating [10,17].

Instead of these traditional techniques, we performed the Cu plating of Glidcop by means of RF-sputtering. The process has a fast deposition rate, and the thickness of the coating can easily be controlled and tailored on the basis of the brazing parameters.

**Figure 6:**
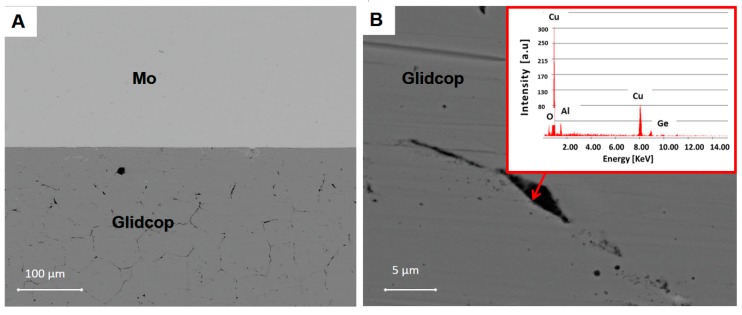
Scanning electron micrographs of (**A**) the cross-section of 1-µm thick Cu- plated Glidcop/Mo joint; (**B**) magnification of a grain boundary in the Glidcop and relative EDS analysis (inset) at about 150 microns from Glidcop/Gemco interface.

This coating process is a complete dry and environmental friendly technique; it is a good process to obtain highly reproducible surface quality, without employing environmentally harmful and toxic substances, thus meeting the Registration, Evaluation, Authorisation and Restriction of Chemicals (EU REACh) regulations [18]. Preliminary experiments were carried out with a 1 µm Cu coating sputtered onto Glidcop; the metallographic examination of the joined Glidcop/Gemco/Mo interface did not show any decohesion at the Cu sputtered/Glidcop interface, and defect-free Gemco/substrate interfaces were observed, as seen in Figure 6. On the other hand, the specimen still exhibited strong intergranular penetration of the braze along the Glidcop grain boundaries (about 200 μm through the thickness). An EDS analysis at the grain boundaries in Glidcop, at about 150 μm from the Gemco/Glidcop interface, seen in Figure 6B, showed the presence of Ge-enriched grain boundary regions in the Glidcop. As a conclusion, the 1 μm Cu sputtered coating was considered as not being effective against the diffusion of Gemco in Glidcop.

**Figure 7:**
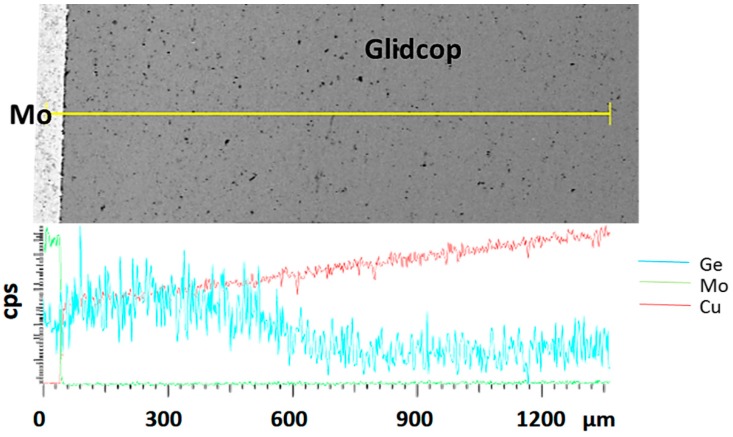
Element distribution (Ge, Cu and Mo) across the 1-µm thick Cu plated Glidcop/braze/Mo joint.

To corroborate this statement, the distribution of the considered elements, obtained from an EDS analysis across the joint region, is shown in Figure 7. The diagram is scaled with respect to the distribution of the elements at the Glidcop/Mo interface. The main braze constituents diffused significantly in the Glidcop up to 600 µm from the interface. On the other hand, Ge-Mo intermetallic phases were able to form at the interface between the Gemco and Mo, according to the phase diagram. Moreover, the EDS analysis detected Ge (up to 4%).

**Figure 8:**
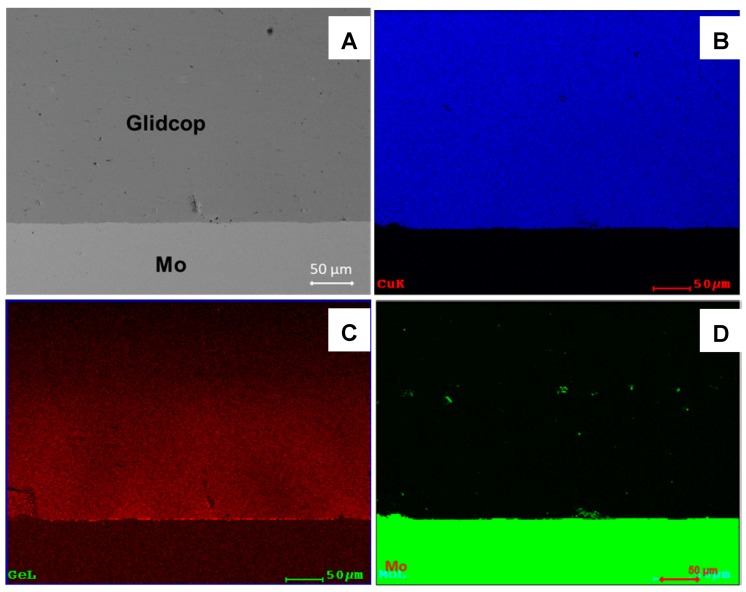
(**A**)SEM cross-section of an 18-µm thick Cu plated Glidcop/Mo joint; (**B**) Cu EDS mapping; (**C**) Ge EDS mapping; (**D**) Mo EDS mapping.

The same analysis was carried out on the joints obtained with a thicker (18 µm) Cu sputtered layer on Glidcop; this value was chosen according to the values reported in literature for electrochemical plating (minimum 15–20 micron). Figure 8 shows the cross-section of a 18-µm thick Cu plated Glidcop/Gemco/Mo joint; no voids are detectable at the braze/adherend interface or along the Glidcop grain boundaries, thus demonstrating that the thicker copper coating successfully limited the diffusion of elements from the braze along the grain boundaries of Glidcop and allowed the formation of a defect-free brazed joint. The EDS element maps reported in Figure 8B–D demonstrate that the Ge was confined to the braze alloy (about 180 µm thick) and did not diffuse into the Glidcop alloy.

In short, the excessive reduction of the filler in the Glidcop, and the consequent formation of detrimental voids in the alloy, can be avoided by depositing a Cu diffusion barrier coating by means of RF-sputtering. Moreover, unlike the commonly adopted electroplating process, this is a simple process that does not require chemicals or lead to blister formation at the plating/substrate interface.

In conclusion, the optimal brazed specimens were manufactured at 980 °C for 1 min, under flowing Ar by using three Gemco foils as brazing material, after having Cu-plated the Glidcop surface by an 18 µm Cu layer by RF-sputtering.

As a consequence, the optimized joints are four components sandwiched, made of Glidcop, thin Cu layer, braze foils, and Mo block.

### Mechanical Tests

Some joints, manufactured using the optimized process were mechanically tested in a single-lap test to determine the apparent shear strength. The fracture did not occur in the joined area, but all the tested samples detached from the fixture, seen Figure 1B; the glue failed for stresses of up to 42 MPa, and no fracturing occurred in the joined samples. Unlike what was observed where a CuZr alloy was joined to Mo using the same braze [4], no plastic deformation occurred in Glidcop, but, since no failure of the joint was observed, this test configuration could not be used to test the apparent shear strength of these joints.

**Figure 9:**
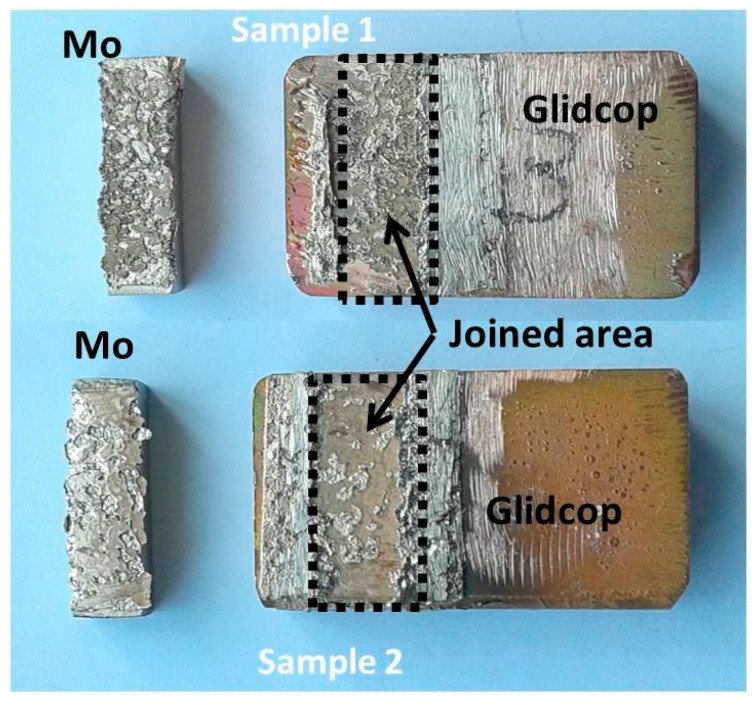
Fracture surface of Mo/Glidcop brazed samples after apparent shear test according to ASTM B898-11.

As a consequence, other brazed specimens were manufactured, according to the ASTM B898-11 standard, seen in Figure 1A, and an average value of 68.5 ± 18.0 MPa was measured. The fracture surface analysis shows that cracks occurred preferentially in the braze and/or at the braze/Glidcop interface, but not at the braze/Mo interface. Figure 9 shows the typical fracture surfaces for a Mo/Glidcop joined sample: the presence of the brazing alloy is evident on both sides.

**Figure 10:**
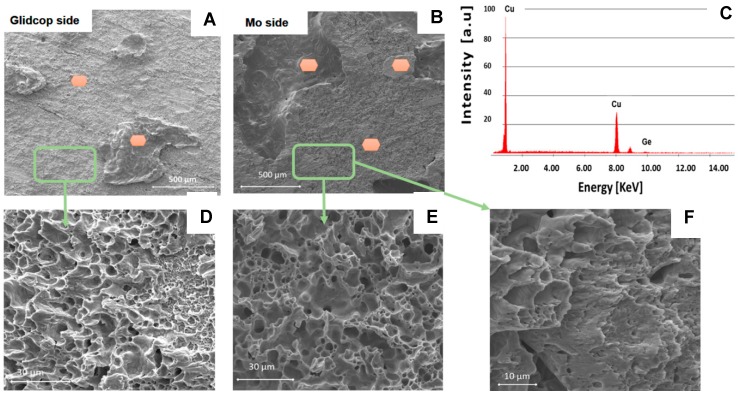
SEM and EDS analysis on Glidcop and Mo fracture surface apparent shear test according to ASTM B898-11; on both surfaces only Cu and Ge have been detected, thus indicating that cracks propagate within the braze and in some areas close to or in Glidcop; the EDS spectrum refers to measurements done in all the orange areas. (**A**) fracture surface of Glidcop side; (**B**) fracture surface of Mo side; (**C**) EDS analysis on orange areas of fracture surfaces shown in (**A**,**B**); (**D**) magnification of fracture surface area on Glidcop side; (**E**) magnification of fracture surface area on Mo side; (**F**) higher magnification of area shown in (**E**).

The fracture mechanism involves coalescence of the microvoids, seen in Figure 10, as in a typical ductile failure. Furthermore, the EDS analysis performed on all the areas marked in Figure 10 only revealed the presence of Cu and Ge contained in the brazing alloy on both of the fracture surfaces; the relative amount of these elements points out that the failure occurred mainly within the braze, thus indicating a lower joint strength between the braze and the Glidcop than the shear strength of the dispersion-strengthened alloy itself. The cohesive behavior of the joined samples after testing demonstrates the good adhesion at the Mo/braze and Glidcop/braze interface.

Hardness tests were carried out in order to investigate the influence of the brazing process on the Glidcop properties; the hardness values of the Glidcop, before and after the brazing process, were 70 ± 2 Hardness Rockwell B (HRB) and 74 ± 1 HRB, respectively. These data show that the brazing process does not result in a significant change in the mechanical properties of the Glidcop, relative to its hardness. 

## 4. Conclusions

The joining of Glidcop AL-25 and Mo has been investigated in detail. A brazing process that involved a commercial Cu-based alloy (Ag free) was studied; it led to microstructurally continuous joints with good shear strength. 

In light of the results of this investigation, the joining parameters were then optimized and Cu plating, by means of sputtering, was used to prevent diffusion of the brazing elements across the joint interface. Unlike the commonly adopted electroplating process, this is a simple process that does not require chemicals or lead to blister formation at the plating/substrate interface.

The optimization of the joining process was based on the number of braze foils and on the study of diffusion of the brazing elements in Glidcop. The sputtering of a copper diffusion barrier on Glidcop, to prevent diffusion of the braze, was effective in reducing but not in completely eliminating the formation of voids and cracks, as a result of the preferential migration of braze along the Glidcop grain boundaries. 

The optimal brazed specimens were obtained at 980 °C, 1 min, under flowing Ar, using 3 Gemco foils as brazing material, after having Cu-plated the Glidcop surface by a 18 µm Cu layer by RF-sputtering. The apparent shear strength (adapted from ASTM B898-11) on the optimized joined samples was about 68 MPa.

## Figures and Tables

**Table 1:** Brazing parameters for several sets of samples; joining temperature fixed at 980 °C, heating rate 1000 °C/h, Ar atmosphere (100 g superimposed weight corresponds to 0.02 MPa nominal pressure).

		Sample 1	Sample 2	Sample 3	Sample 4	Sample 5	Sample 6	Sample 7	Sample 8
**Set up**	Number of Brazing Foils	3	3	3	1	1	3	3	3
	Superimposed Weight (100 g)	yes	yes	no	yes	no	yes	yes	yes
**Process parameters**	Dwelling Time (min)	1	5	5	5	5	10	15	30

## References

[1] Aghamiri S.M.S., Oono N., Ukai S., Kasada R., Noto H., Hishinuma Y., Murogac T. (2018). Microstructure and mechanical properties of mechanically alloyed ODS copper alloy for fusion material application. Nucl. Mater. Energy.

[2] Bertarelli A., Aberle O., Assmann R., Chiaveri E., Kurtyka T., Mayer M., Perret R., Sievers P. The mechanical design for the LHC collimators. Proceedings of the EPAC.

[3] Bertarelli A., Dallocchio A., Garlasché M., Gentini L., Gradassi P., Guinchard M., Redaelli S., Rossi A., Sacristan de Frutos O., Quaranta E. Novel materials for collimators at lhc and its upgrades. Proceedings of the 54th ICFA Advanced Beam Dynamics Workshop on High-Intensity, High Brightness and High Power Hadron Beams, HB2014.

[4] Salvo M., Casalegno V., Rizzo S., Ferraris M., Izquierdo G., Heikkinen S., Sgobba S., Taborelli M. (2010). Brazing of Mo to a CuZr alloy for the production of bimetallic raw materials for the CLIC accelerating structures. J. Mater. Process. Technol..

[5] Ljvak R.J., Frost H.M., Zocco T.G., Kennedy J.C., Hobbs L.W. (1986). Promising copper alloys for high heat load applications in neutron environments. J. Nucl. Mater..

[6] Braham C., Coppola R., Nardi C., Valli M. (2005). High temperature stresses in brazed Glidcop/W model structures of interest for ITER divertor technology. Fusion Eng. Des..

[7] Yadav D.P., Kaul R., Ram Sankar P., Kak A., Ganesh P., Shiroman R., Singh R., Singh A.P., Tiwari P., Abhinandan L. (2012). A study on brazing of Glidcop^®^ to OFE Cu for application in Photon Absorbers of Indus-2. J. Phys. Conf. Ser..

[8] Nishi H., Muto Y., Sato K. (1994). Solid-state diffusion bonding of alumina dispersion-strengthened copper to 316 stainless steel. J. Nucl. Mater..

[9] Nishi H., Kikuchi K. (1998). Influence of brazing conditions on the strength of brazed joints of alumina dispersion-strengthened copper to 316 stainless steel. J. Nucl. Mater..

[10] Chen S., Bao T., Chin B.A. (1996). Braze joints of dispersion strengthened copper. J. Nucl. Mater..

[11] Chen S., Liu J.Y., Chin B.A. (1994). Effect of alumina strengthening particles on brazed joints of GlidCop Al-15 copper alloy. J. Nucl. Mater..

[12] (2013). ASTM D905-08 (2013) Standard Test Method for Strength Properties of Adhesive Bonds in Shear by Compression Loading.

[13] (2016). ASTM B898—11 (Reapproved 2016) Standard Specification for Reactive and Refractory Metal Clad Plate.

[14] Tomsia A.P., Saiz E., López-Esteban S., Benhassine M., De Coninck J., Rauch N., Rühle M. (2007). Wetting of metals and glasses on Mo. Int. J. Mater. Res..

[15] Toter W., Sharma S. (2004). Analysis of Gold-Copper Braze Joints in Glidcop^®^ for UHV Components at the Advanced Photon Source. http://citeseerx.ist.psu.edu/viewdoc/summary?doi=10.1.1.693.9571.

[16] Wang J.S., Leinenbach C., Jacot A. (2010). Thermodynamic assessment of the Cu–Ge binary system. J. Alloys Compd..

[17] Sgobba S. (2006). Materials of High Vacuum Technology: An Overview. CAS-CERN Accelerator School and ALBA Synchrotron Light Facility: Course on Vacuum in Accelerators.

[18] (2013). Regulation (EC) No. 1907/2006 of the European Parliament and of The Council of 18 December 2006 Concerning the Registration, Evaluation, Authorisation and Restriction of Chemicals (REACH), Establishing a European Chemicals Agency.

